# Inter-population Differences in Retrogene Loss and Expression in Humans

**DOI:** 10.1371/journal.pgen.1005579

**Published:** 2015-10-16

**Authors:** Michał Kabza, Magdalena Regina Kubiak, Agnieszka Danek, Wojciech Rosikiewicz, Sebastian Deorowicz, Andrzej Polański, Izabela Makałowska

**Affiliations:** 1 Department of Bioinformatics, Institute of Molecular Biology and Biotechnology, Faculty of Biology, Adam Mickiewicz University, Poznań, Poland; 2 Institute of Informatics, Faculty of Automatic Control, Electronics and Computer Science, Silesian University of Technology, Gliwice, Poland; National Institute of Genetics, JAPAN

## Abstract

Gene retroposition leads to considerable genetic variation between individuals. Recent studies revealed the presence of at least 208 retroduplication variations (RDVs), a class of polymorphisms, in which a retrocopy is present or absent from individual genomes. Most of these RDVs resulted from recent retroduplications. In this study, we used the results of Phase 1 from the 1000 Genomes Project to investigate the variation in loss of ancestral (i.e. shared with other primates) retrocopies among different human populations. In addition, we examined retrocopy expression levels using RNA-Seq data derived from the Ilumina BodyMap project, as well as data from lymphoblastoid cell lines provided by the Geuvadis Consortium. We also developed a new approach to detect novel retrocopies absent from the reference human genome. We experimentally confirmed the existence of the detected retrocopies and determined their presence or absence in the human genomes of 17 different populations. Altogether, we were able to detect 193 RDVs; the majority resulted from retrocopy deletion. Most of these RDVs had not been previously reported. We experimentally confirmed the expression of 11 ancestral retrogenes that underwent deletion in certain individuals. The frequency of their deletion, with the exception of one retrogene, is very low. The expression, conservation and low rate of deletion of the remaining 10 retrocopies may suggest some functionality. Aside from the presence or absence of expressed retrocopies, we also searched for differences in retrocopy expression levels between populations, finding 9 retrogenes that undergo statistically significant differential expression.

## Author Summary

Many retrogenes, long considered to be genomic “junk”, were recently revealed as vitally important. Retroposition plays an important role in shaping differences between species, populations and individuals. Variation may result either from new retroposition events and/or retrocopy loss, as well as from differences in retrocopy expression. Genome analysis of 1092 individuals from various populations and transcriptomes from 50 individuals, revealed differences between populations in the frequency of transcriptionally active ancient retrocopy loss, as well as differences in retrocopy expression levels. Overall, these results provide new insights into the genomic events of inter-population variation, which has not been evaluated much with respect to gene loss.

## Introduction

Retro(pseudo)genes are gene copies that originate through the process of reverse transcription of mRNAs and their subsequent insertion into the genome. This process, called retroposition is usually mediated by enzymes provided by retrotransposable elements and creates intronless copies of existing genes [1]. Due to the lack of promoter sequence of their parental genes, retrocopies were long assumed to be nonfunctional and simply “junk DNA”. However, multiple findings challenged that view and a few mechanisms were proposed for retrogene transcription [2]. Functional retrocopies (retrogenes) may have an intact open reading frame and thus be protein-coding, but can also act as long noncoding RNAs [3], sources of short interfering RNAs [4] and microRNA sponges [5]. The loss of parental regulatory sequences and potential replacement with those recruited from the new locus of integration is believed to be the most possible reason that retrogenes are able to undergo neofunctionalization more often than other types of gene copies [6] and take part in the shaping of lineage- and species-specific features [7]. As recently shown, retrogenes can also replace their parental genes [8]. Apart from their functionality, retrocopies are also useful phylogenetic markers, providing insight into such evolutionary processes as sex chromosome origination [9] or changes in retrotransposable element activity and germ line gene expression [2].

In recent years, numerous studies have shown that many retrogenes are vitally important and a number of them are involved in diseases. A good example is the *RHOB* gene (ras homolog gene family member B [MIM: 165370]), a tumor suppressor that belongs to the Rho GTPases family [10]. A mutation in another retrogene, *RNF113A* (ring finger protein 113A [MIM: 300951]), results in trichothiodystrophy [11]. On the other hand, insertion of the *PPIA* cDNA (peptidyl-prolyl isomerase A [MIM: 123840]) into the *TRIM5* gene (tripartite motif-containing protein 5 [MIM: 608487]) confers resistance to HIV in owl monkeys [12].

Retroposition gives rise to considerable genetic variation between individuals. Recent developments in sequencing technology allow researchers to move beyond the analysis of individual genomes from model organisms to the study of retrocopies within a population. Large-scale sequencing projects, such as the 1000 Genomes Project [13] enable the exploration of differences in copy-number variation within human populations. Three recent studies [14–16] on the retrocopy repertoire in human populations revealed a total of 208 polymorphic retrocopies [17] called retroduplication variations (RDVs). In addition, two of them [14, 15] aided in reconstructing the phylogenetic tree of human populations. Thus, proving the value of RDV polymorphisms as genomic markers for population history.

Despite these advances, many questions remained unanswered. Current methods of discovering novel retropositions, (i.e. not annotated on the reference genome), utilize paired-end reads and require at least one read from the pair to map to the parental gene of the retrocopy. As a result, this approach allows only for the detection of retrocopies that originated relatively recently in evolutionary history and show little sequence divergence compared to their parental genes. Evolutionary events that happened earlier in the human lineage and resulted in currently observed RDVs have been less explored. Moreover, we know surprisingly little about the functional aspects of different kinds of retrogene polymorphisms. Single nucleotide polymorphisms located in retrogene promoters and regulatory sequences might result in different levels of retrogene expression between individuals and populations. Presence or absence of a functional retrocopy might have far reaching biological implications. Due to lack of reliable experimental data, these issues have thus far been ignored; yet, this will most certainly change in the near future.

In this study, we focused on aspects not covered by aforementioned publications. We used the results of Phase 1 from the 1000 Genomes Project to investigate variation in loss of ancient (i.e. shared with other primates) retrocopies among human populations. In addition, we utilized RNA-Seq data from the Ilumina BodyMap project as well as data from the lymphoblastoid cell lines of 50 individuals provided by the Geuvadis Consortium [18] to examine variation in the expression level of retrocopies.

## Results

### Identification of RDVs from known retrocopies in human populations

To analyze retrocopy number variation resulting from retrocopy loss in human populations we used 4,927 human retrocopies from RetrogeneDB [19] and the genomic data from Phase 1 of the 1000 Genomes Project [13]. The genomic data consisted of whole genome sequencing of 1,092 individuals from 14 populations. By mapping human retrocopies to genomic variants detected during Phase 1 of the 1000 Genomes Project, we identified 214 indels that affected 190 retrocopies. To be more explicit, 190 retrocopies annotated in the human reference genome were at least partially missing (i.e. at least 100 bp of the retrocopy sequence was deleted) in some of analyzed genomes. Next, we calculated allele frequencies of detected indels in 14 available populations (S1 Table and RVD Maps online material). Out of all identified indels involving retrocopies, 67 were population specific and 11 were observed in all investigated populations. Forty-eight indels were relatively widespread and affected 6–8 populations, and 88 occurred in only 2–5 populations.

**Fig 1:**
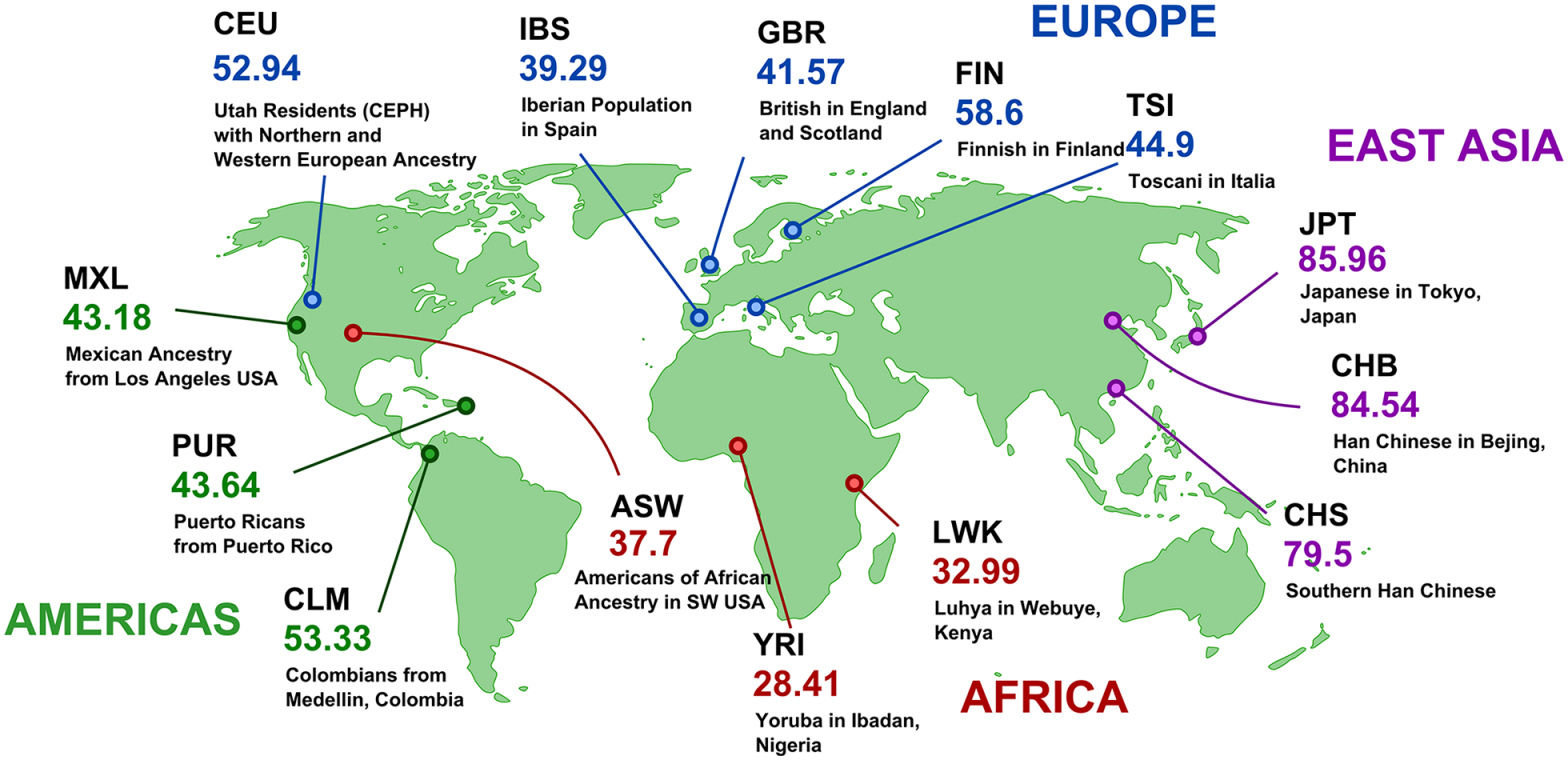
Frequencies of retro_hsap_1441 absence in different human populations. Maps for all retrocopies are available at http://rhesus.amu.edu.pl/RetrogeneMaps/.

Indels that were detected only rarely in populations have, with some exceptions, low frequencies and are observed in 0.5%–2% of alleles. This may suggest that these indels represent either relatively new deletions or deleterious deletions, and thus, were subjected to negative selective pressure. The highest rate of absence was observed in the case of retrogene retro_hsap_1441 (Fig 1). This retrogene is present in about 20% of alleles in Asian populations, in about 50% of the alleles in European and American populations, and in about 70% in populations with African ancestry. The most likely explanation for this phenomenon is the emergence of a new retroposition in Africa, which spread to other continents, or alternatively, a deletion that originated in Asia. However, based on available data we cannot distinguish between these two scenarios.

### Detection of the loss of ancestral, expressed retrocopies

Due to the methodology applied, previous studies [14–16], indicated that the majority of retrocopy polymorphisms resulted from novel retropositions. In this study, we focused on the identification of polymorphisms that resulted from a retrocopy deletion. We can assume that the lack of a retrogene observed at low frequency and only in selected populations represents a deletion. However, when absence of the retrogene is common across populations and detected at relatively high frequency, the polymorphism may be a result of either deletion or new retroposition. Therefore, to identify retrocopy deletion events, we performed comparative analysis across fourteen Eutherian species in order to identify retrocopy orthologs that originated prior to human speciation.

**Fig 2:**
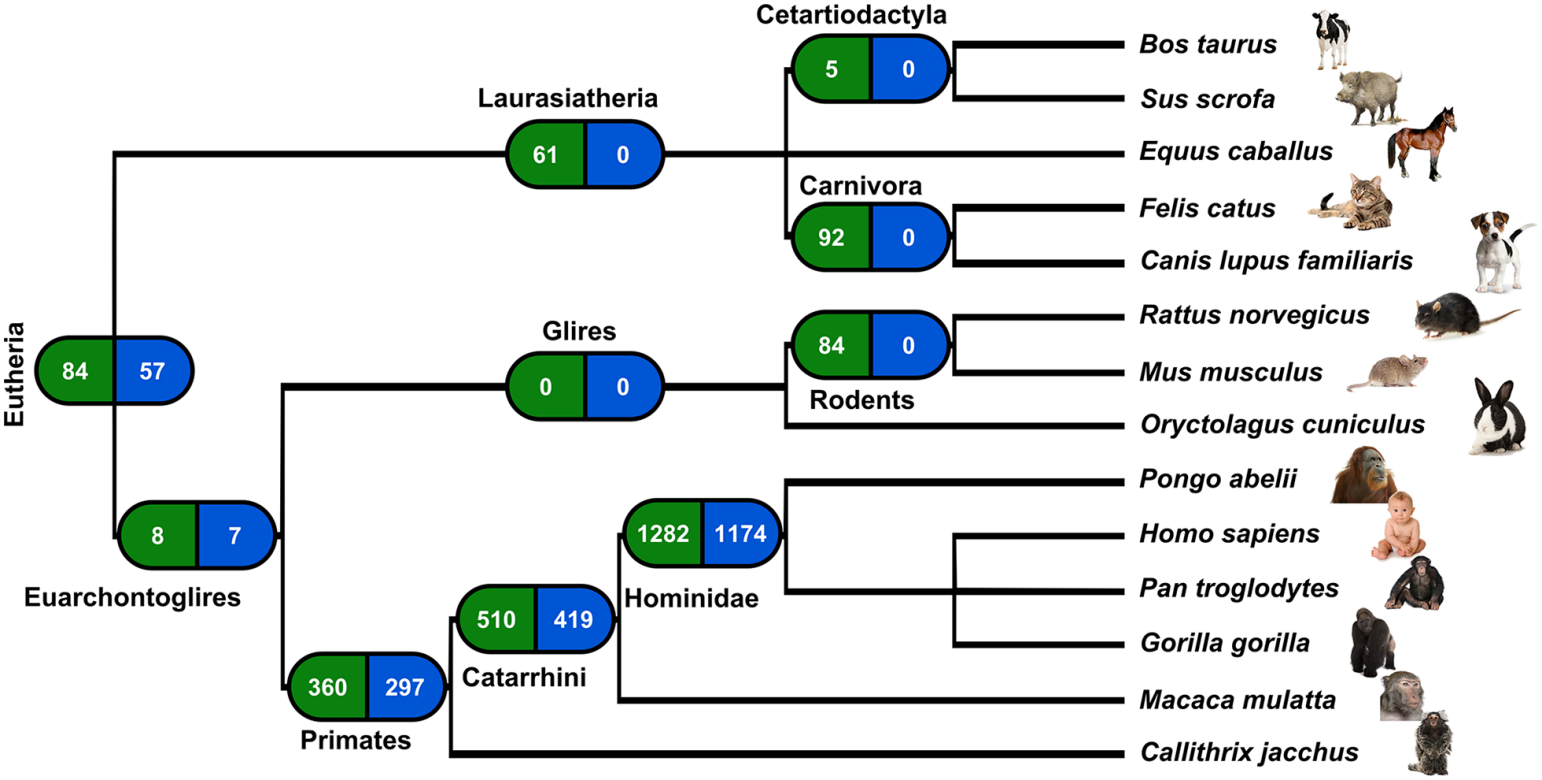
Retroposition events in Eutheria. Left boxes show number of retrocopies originated in a given lineage ancestors’ genome; right boxes show number of retrocopies detected in the human genome.

In the case of retroposition, a reciprocal sequence similarity search is not sufficient to establish orthology, since many genes undergo independent retroduplication in different species [20]. Hence, in order to pinpoint orthologous retrocopies we used information concerning the location of retrocopies identified by us in mammalian genomes [19] and mapped them on the genomic sequences alignments using Ensembl release 73. First, we checked for orthologous genes in major mammalian lineages. We classified a retrocopy as common for a lineage if it was observed in any two species from this lineage. The requirement of retrocopy presence in a minimum of two but not necessarily all genomes is based on the assumption that the probability of independent retroposition of the same gene at the same location is close to zero. Using this approach, we identified as many as 1,282 retroposition events that took place in the Hominidae ancestor genome. As many as 510 retropositions are common to Catarrhini, and 360 to primates. In comparison, we did not detect any retrocopies common to Glires and only 84 shared by mouse and rat (Fig 2). This analysis confirms a burst of retroposition in Primates and shows that it was especially intensive in Hominidae [7].

While considering only human retrocopies, we found that 1,954 were present in at least one other genome. From these, 1,174 originated in the common ancestor of Hominidae and 57 are very ancient, as they are present in at least one genome of analyzed Eutheria other than Eurchontoglires (Fig 2). From the set of polymorphic retrocopies, 68 were among those we identified as ancestral. Therefore, with high confidence we can say that the polymorphism of these 68 retrocopies resulted from deletion and not from a new retroposition event.

**Fig 3:**
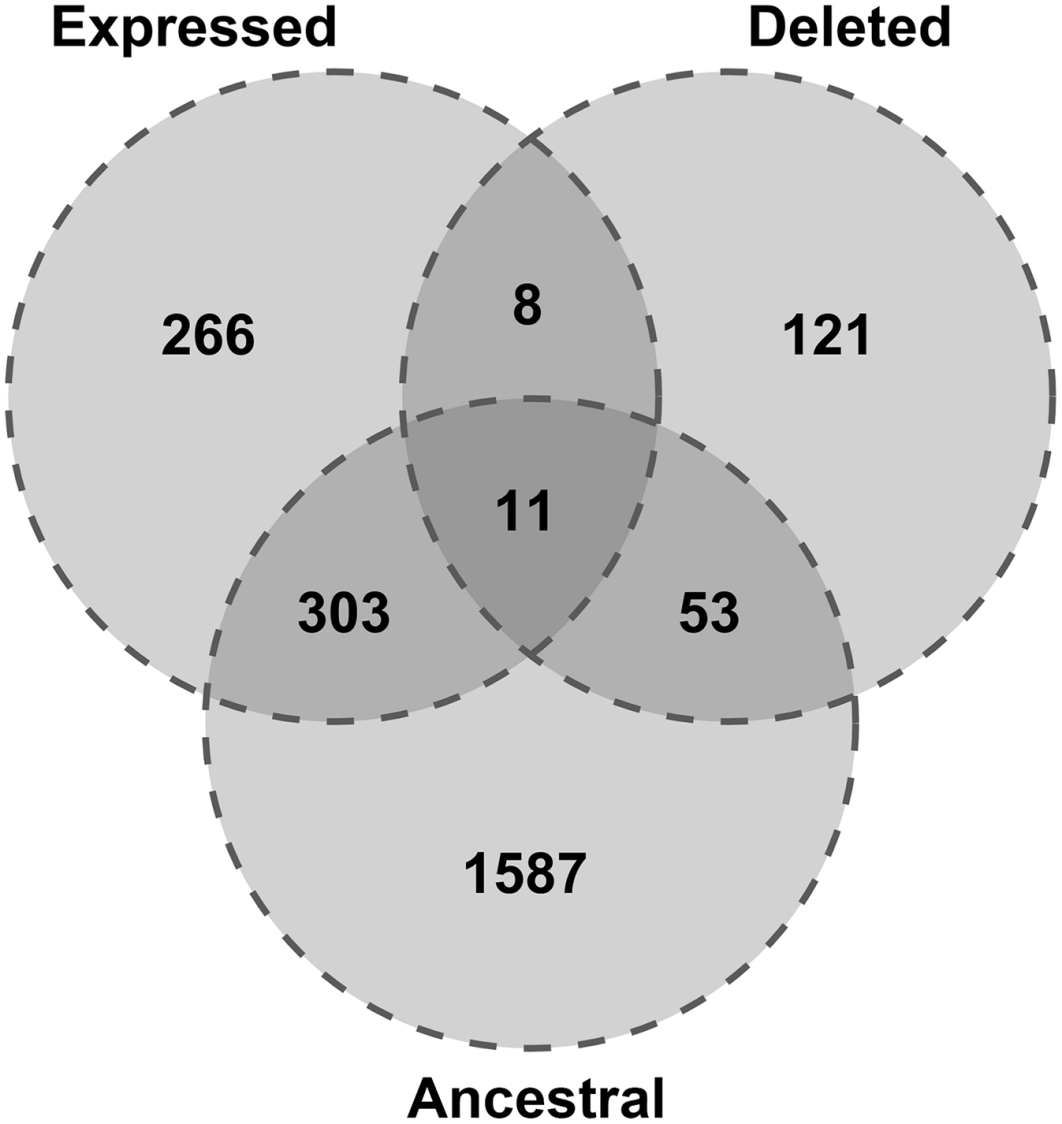
Expression and conservation of retrocopies deleted relative to the reference genome.

Most retrocopies are non functional and therefore, their loss is in most cases neutral. However, many retrocopies are expressed and this, together with conservation may indicate some functionality. To assess retrocopy expression, we used RNA-Seq samples from ten individuals selected from five different populations (CEU, GBR, FIN, TSI, YRI) derived from the Geuvadis RNA sequencing project [18], as well as data for 16 human tissues from the Illumina Bodymap 2.0 project. To distinguish between parental gene and the retrocopy, only uniquely mapped reads were considered. As expressed we considered a retrocopy with a normalized expression value of at least 1 RPM (reads per million mapped reads) in at least one sample, either one library from the Body Map project or one individual from the Geuvadis RNA sequencing project. These criteria were met by 588 retrocopies, and 11 of them were also ancestral and absent in some individuals (Fig 3).

### Characterisation of ancestral, expressed retrocopies undergoing deletion

We calculated Fisher's exact test to assess the relationship between expression, ancestrality and retrogene loss. The obtained p-values were all higher than 0.05 and therefore, neither conserved nor expressed retrocopies are less likely to be polymorphic. Most ancestral and expressed retrocopies are deleted in only a small fraction (0.5–2%) of alleles from certain populations, suggesting that their deletions are either relatively new or slightly deleterious; thus, were subjected to negative selective pressure (Table 1).

**Table 1:** Frequencies of ancestral and expressed retrogene loss in human populations.

Retrocopy	Indel Length	Population													
		African			Ad Mixed American			European					East Asian		
		ASW	YRI	LWK	MXL	PUR	CLM	CEU	IBS	GBR	FIN	TSI	JPT	CHB	CHS
**retro_hsap_88**	1272	0	0	3.09	0.76	0	1.67	0.59	0	0	1.08	0.51	1.69	1.03	0
**retro_hsap_385**	6257	0	0	0	0	1.82	0.83	0	0	0	0	0	0	0	0
**retro_hsap_477**	16238	0	0	0	0	0	0	0	0	0	0	0	0	1.03	0.5
**retro_hsap_1068**	46133	0.82	0	0	0	0	0	0	0	0	0	0.51	0	0	0
**retro_hsap_1629**	5062	0	0	0	0	0	0	0	0	0.56	0	0	0	0	0
**retro_hsap_1793**	4082	0.82	0.57	1.03	0	1.82	0	0	0	0	0	0	0	0	0
**retro_hsap_2011**	36761	0	0	0	0	0	0	0	0	0	1.08	0	0	0	0
**retro_hsap_2905**	6836	0	0	0	0	0	0	0	0	0	0	0.51	0	0	0
**retro_hsap_3468**	2181	0.82	0	0	3.03	3.64	5	5.29	3.57	11.24	3.76	5.61	0	0	0
**retro_hsap_4123**	9011	0.82	0.57	1.03	0	0	0	0	0	0	0	0	0	0	0
**retro_hsap_4873**	1260	15.31	11.28	15.07	0.99	1.22	0	0	0	0	0	0	0	0	0

To confirm and evaluate the expression of 11 transcriptionally active ancestral retrocopies undergoing deletion, we decided to use regular PCR followed by quantitative PCR (real-time PCR). In the case of two retrogenes (retro_hsap_4873 and retro_hsap_2011) we were not able to identify primers suitable for qPCR; therefore, we selected primers for standard PCR only. We ran standard PCR in 16 cDNA libraries from various organs, and sequenced amplification products to confirm primer specificity. Sequencing was necessary, as primers specific for a given retrogene could still amplify RNA expressed from a parental gene. Indeed, in the case of one retrocopy (retro_hsap_88) products were not always specific (S1 Fig). In addition, primers designed for two other retrocopies (retro_hsap_2905 and retro_hsap_4123) formed dimers. Finally, primers for retro_hsap_477 functioned only at a much lower temperature than is required for qPCR. For these four retrocopies, as in the case of retro_hsap_4873 and retro_hsap_2011, we were not able to identify alternative pairs of primers meeting qPCR requirements. Nevertheless, we managed to confirm the expression of all 11 retrocopies by regular PCR (we found working primers for longer products). Sequences of all primers are provided in S2 Table.

**Fig 4:**
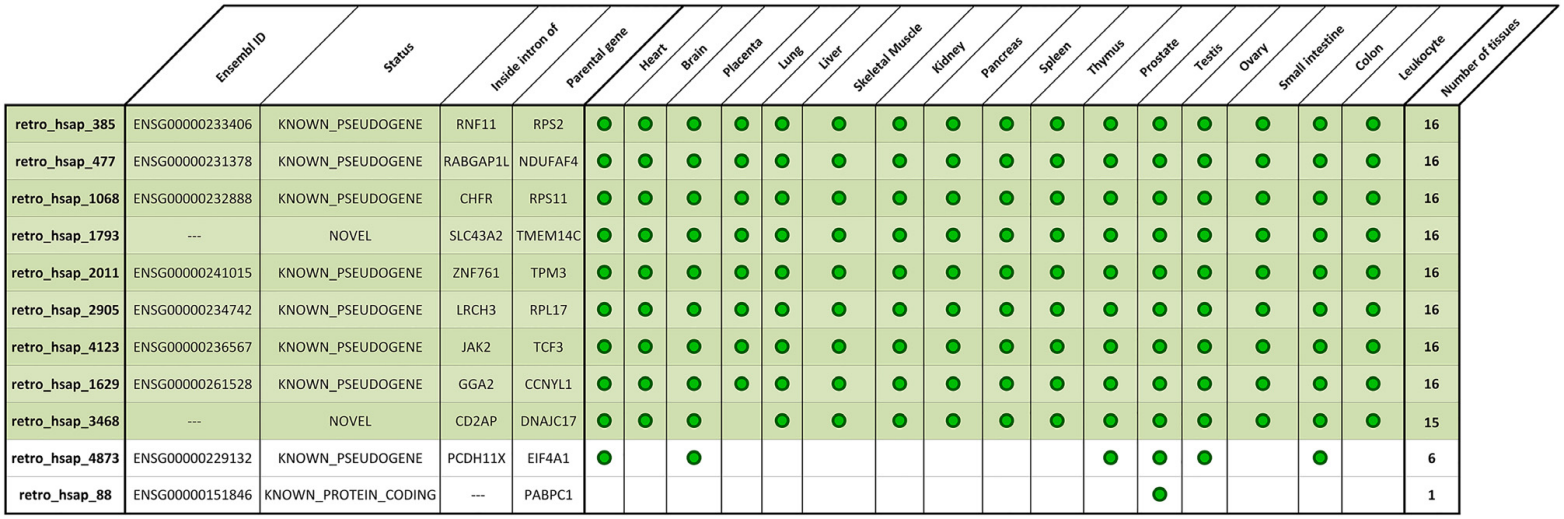
Expression pattern of deleted expressed retrocopies based on standard PCR.

The analysis showed that eight retrogenes are expressed in all sixteen analyzed libraries and one (retro_hsap_3468) is expressed in all but lung. In contrast, retro_hsap_88 is expressed only in testis. In the case of retro_hsap_4873, we could with high confidence, confirm expression in six organs (Fig 4 and S1 Fig).

**Fig 5:**
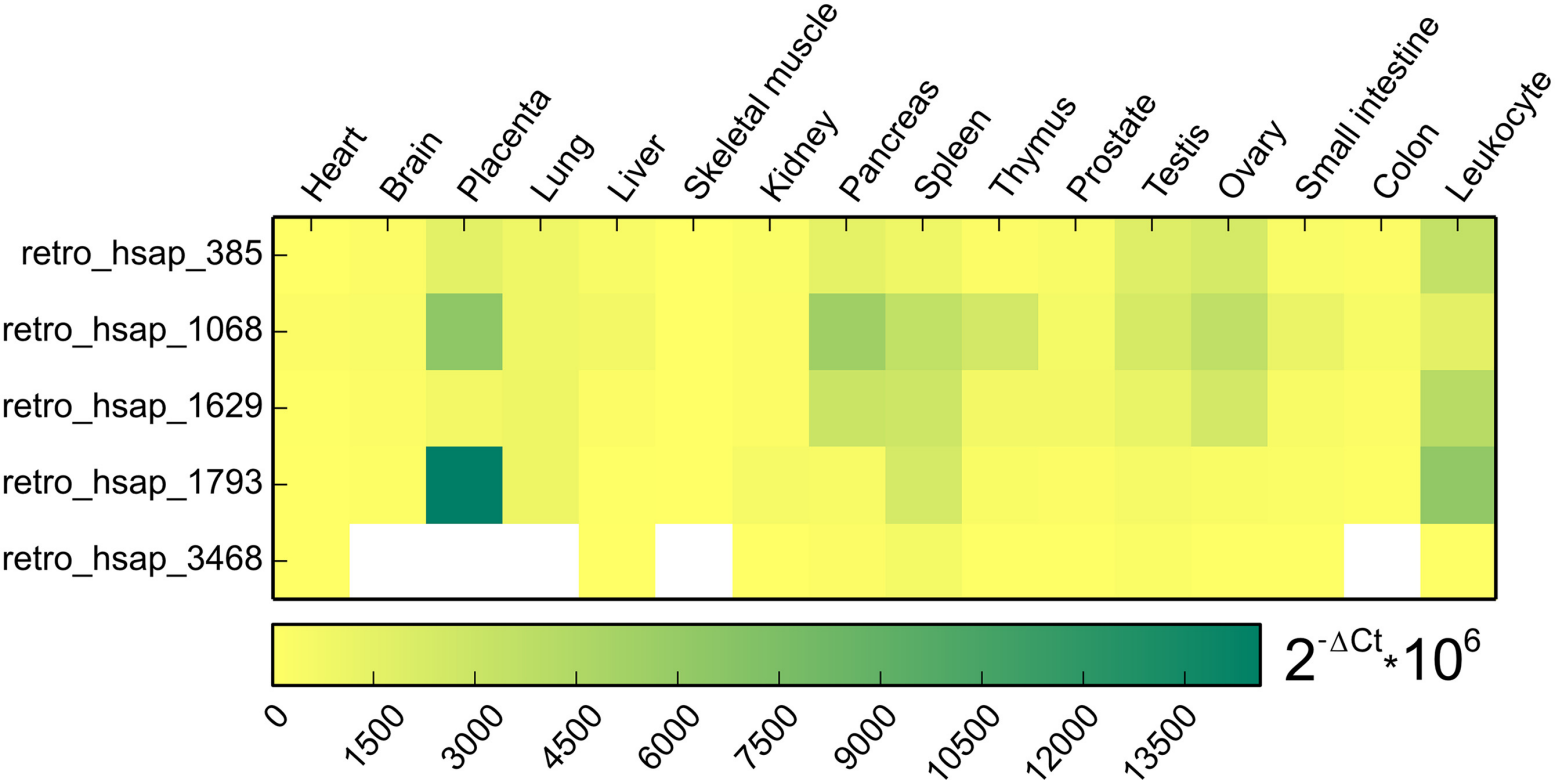
Heat map representing expression pattern of five retrogenes based on qPCR. White color indicates tissues with no significant expression level (Ct > 32).

Due to the above-mentioned reasons, qPCR could be performed on only five retrocopies. It revealed low-level expression in nearly all organs. High expression is observed only for retro_hsap_1793 in placenta and some retrocopies show moderate expression in pancreas, spleen, testis, ovary, and leukocyte (Fig 5). In addition, although retro_hsap_3468 exhibits expression in all but one organ when amplified by standard PCR, qPCR did not yield significant products in five libraries (Fig 5).

Based on our earlier retrocopy analysis [19], we determined that only one out of 11 lost expressed retrocopies (retro_hsap_88) is annotated as a single exon gene (Ensembl: ENSG00000151846) coding for the cytoplasmic 3 poly(A) binding protein (*PABPC3* [MIM: 604680]). Eight retrogenes are annotated in the Ensembl database as processed pseudogenes, and two (retro_hsap_3468 and retro_hsap_1793) are novel, i.e. identified by us [19] and are not annotated in the Ensembl or other databases (Fig 4). Three retrocopies originated from ribosomal protein coding genes, which is not surprising as these genes yielded an exceptionally high number of retrocopies [21].

The function of these 11 retrogenes is not known, with the exception of retro_hsap_88, which codes for a known protein. Only three of the remaining 10 retrocopies have conserved open reading frames (ORF), i.e. there are no stop codons or frame shifts over the entire retrocopy-parental gene alignment. Interestingly, these three retrocopies are those originated from genes encoding for ribosomal proteins.

**Fig 6:**
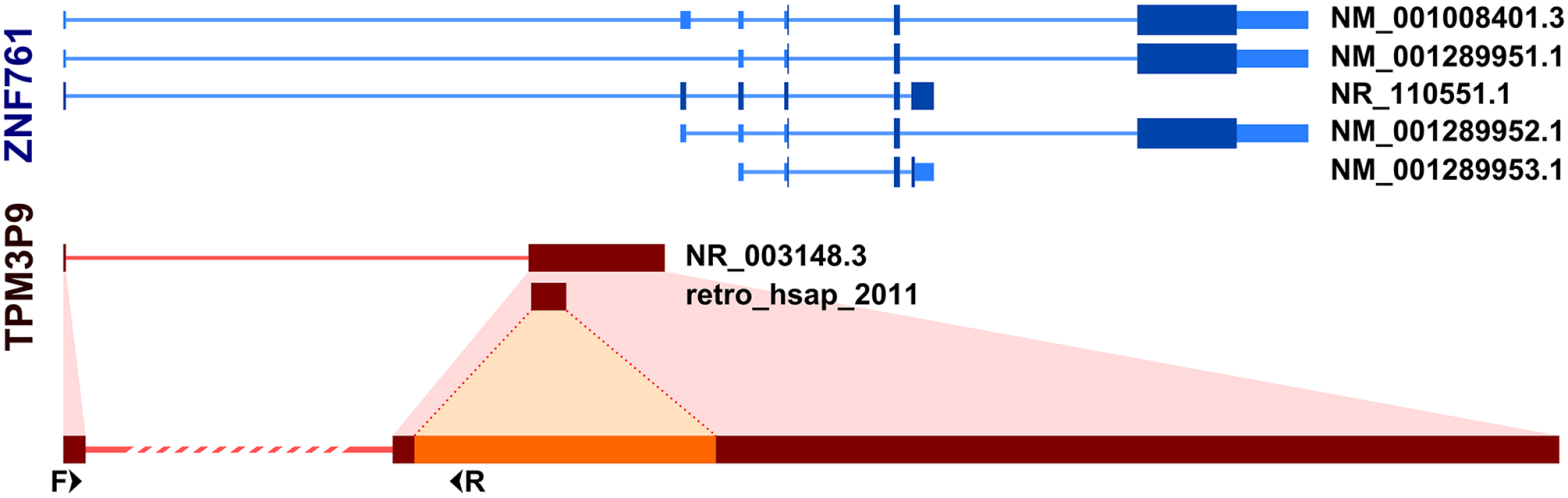
Incorporation of host gene exon by retrogene retro_hsap_2011. Splice variants of *ZNF761* gene (upper part) and *TPM3P9* retrogene (lower part). F and R refer to forward and reverse primer binding sites, respectively.

With one exception (retro_hsap_88), all analyzed retrocopies are located in the introns of other genes; which is quite typical for retrocopies. Interestingly, retrogene located in the first intron of the zinc finger protein 761 gene (*ZNF761*), retro_hsap_2011 (annotated in the Gene database as *TPM3P9*), exapted the first exon of its host gene, according to annotations. This modification allowed the retrocopy to acquire a regulatory machinery, and consequently, the ability to be expressed, which we confirmed by standard PCR. In addition, part of the *ZNF761* intron was incorporated as part of a retrogene exon (Fig 6). Another possible scenario is that a retrogene was incorporated into an existing two-exon splice variant. In any event, this retrocopy represents yet another example of the formation of new genes via retroposition followed by structural evolution [6, 22].

### Differences between populations in retrogenes expression

Evolutionary changes involve more than just SNPs or mutations in the coding sequence. The differences between species, populations or individuals may also result from mutations in the regulatory regions, and can result in varying expression levels of an affected gene. Therefore, in addition to the analysis of retrocopy absence, we also analyzed retrocopy expression level differences between populations. To perform this analysis we utilized the DESeq2 differential expression package [23] and retrocopy expression estimations for lymphoblastoid cell lines from 50 individuals within five populations (CEU, GBR, FIN, TSI, YRI); 10 individuals per population. The analysis was performed on all 4,927 retrocopies downloaded from RetrogeneDB. In general, the differences detected were not significant. However, in nine retrogenes, we observe notable fold changes (Table 2). As expected, most differences occurred between European and African populations, although the British population was revealed to be somehow distinct from populations originating from continental Europe. The most frequently observed difference is between British and Yoruba populations. Three retrocopies have significantly higher expression levels in individuals from the British population. One of them, retro_hsap_3129, is also exhibits a higher expression in the Finnish population when compared with Yoruba. Two retrogenes demonstrate a higher level of expression in Youruba than in the British population. The British population has one retrocopy expressed at a higher level than the Finnish population, and one expressed at lower levels compared to Utah residents of European ancestry. The remaining differences between populations include an additional retrogene in which the expression is significantly higher in Finnish compared to Yoruba, and one retrocopy with lower expression in individuals from Toscani when compared to individuals from Yoruba.

**Table 2:** Retrocopies undergoing differential expression between analyzed populations.

Retrocopy	Parental gene	Conserved ORF	Other tissues	Reference population	Tested population	Fold change
**retro_hsap_108**	RHOA	Yes	All 16 tissues	GBR	FIN	0.43
**retro_hsap_1259**	RCN1	No	Heart, lung, ovary, prostate	GBR	YRI	0.47
**retro_hsap_1692**	TMEM254	Yes	-	GBR	YRI	2.10
**retro_hsap_1750**	TAF5L	No	Lymph node	CEU	GBR	2.20
**retro_hsap_2310**	RPL23A	No	-	FIN	YRI	0.49
**retro_hsap_2684**	PDCL3	No	-	GBR	YRI	0.41
**retro_hsap_3129**	MYL12B	Yes	-	GBR	YRI	0.44
**retro_hsap_3129**	MYL12B	Yes	-	FIN	YRI	0.48
**retro_hsap_3265**	RPL10	Yes	Lung, skeletal muscle	TSI	YRI	2.29
**retro_hsap_4127**	RPL23A	No	-	GBR	YRI	2.58

### The detection of retrocopies absent from the reference genome

We developed a new methodology in order to identify novel (i.e. not annotated in the reference genome) retrocopies deleted in some individuals. We performed de novo assembly of unmapped reads from 500 individual genomes included in Phase 1 of The 1000 Genomes Project. This yielded 6,911 contigs, 2,901 of which were singletons detected in only one individual. We excluded sequences that showed similarity to human genome patches, alternative assemblies, as well as genomic contaminations. 1,233 (17.8%) sequences were filtered out as bacterial and viral contaminations. Strong similarity to GRCh37.p10 and HuRef human genome assemblies was observed in 861 (12.4%) and 1,767 (25.6%) respectively. Finally, we applied the retrocopy identification pipeline used previously in RetrogeneDB [19] to detect novel retrocopies. As a result, five novel retrocopies were identified. Four of the identified retrocopies were found in only a few individuals (1 to 10). However, retrocopy rdn1 was relatively frequent and was identified in 40 individuals (Table 3).

**Table 3:** Retrocopies absent in the reference genome.

Retrocopy	rdn1	rdn2	rdn3	rdn4	rdn5
**Length (bp)**	373	459	331	338	460
**Parental gene**	C9orf85	RPL21	PTGES3	RARRES2	RPL6
**Deletion site in reference genome**	chr3: 66287175–66287285	chr16: 90107060–90107061	chr9: 104121215–104121216	---	---
**Identity (protein level)**	66.67%	72.02%	52.21%	61.19%	82.47%
**Number of individuals**	40	9	10	1	2

All contigs containing retrocopies show strong (at least 99% identity) similarity to some human clone sequences in the BLAST nr/nt database, suggesting that they were sequenced, but were not included in any major human genome assembly. Comparison of detected retrocopies with the results of previous studies [14–16] reveals that these retrocopies are in fact, novel.

**Fig 7:**
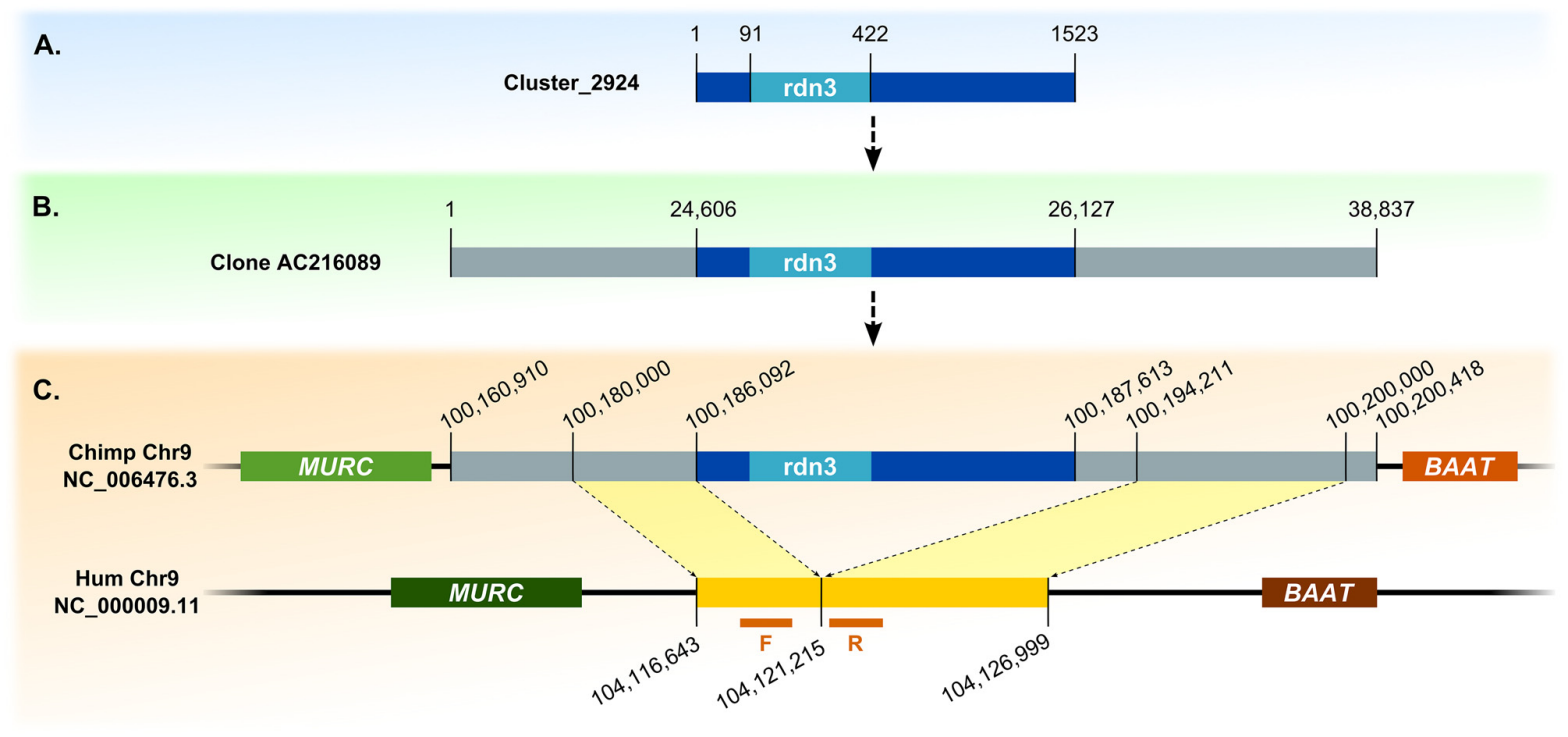
Detection of novel retrocopy deletion sites, example of retrocopy rdn3. (A) Contig and BAC containing its sequence. (B) Alignment with chimpanzee genome. (C) Identification of indel site. F and R refer to forward and reverse primers designed for examined indel site in human genome, respectively.

Detected retrocopies show significant divergence between their sequences and sequences of their parental genes, indicating old retroposition events. We were able to find the orthologs of discovered retrocopies in the chimpanzee and/or gorilla genomes, confirming that they are indeed, ancestral retroduplications that were subsequently lost during human evolution. This analysis also allowed us to determine exact locations and sizes of deletions for retrocopies rdn1, rdn2 and rdn3 (Table 3 and Fig 7).

**Fig 8:**
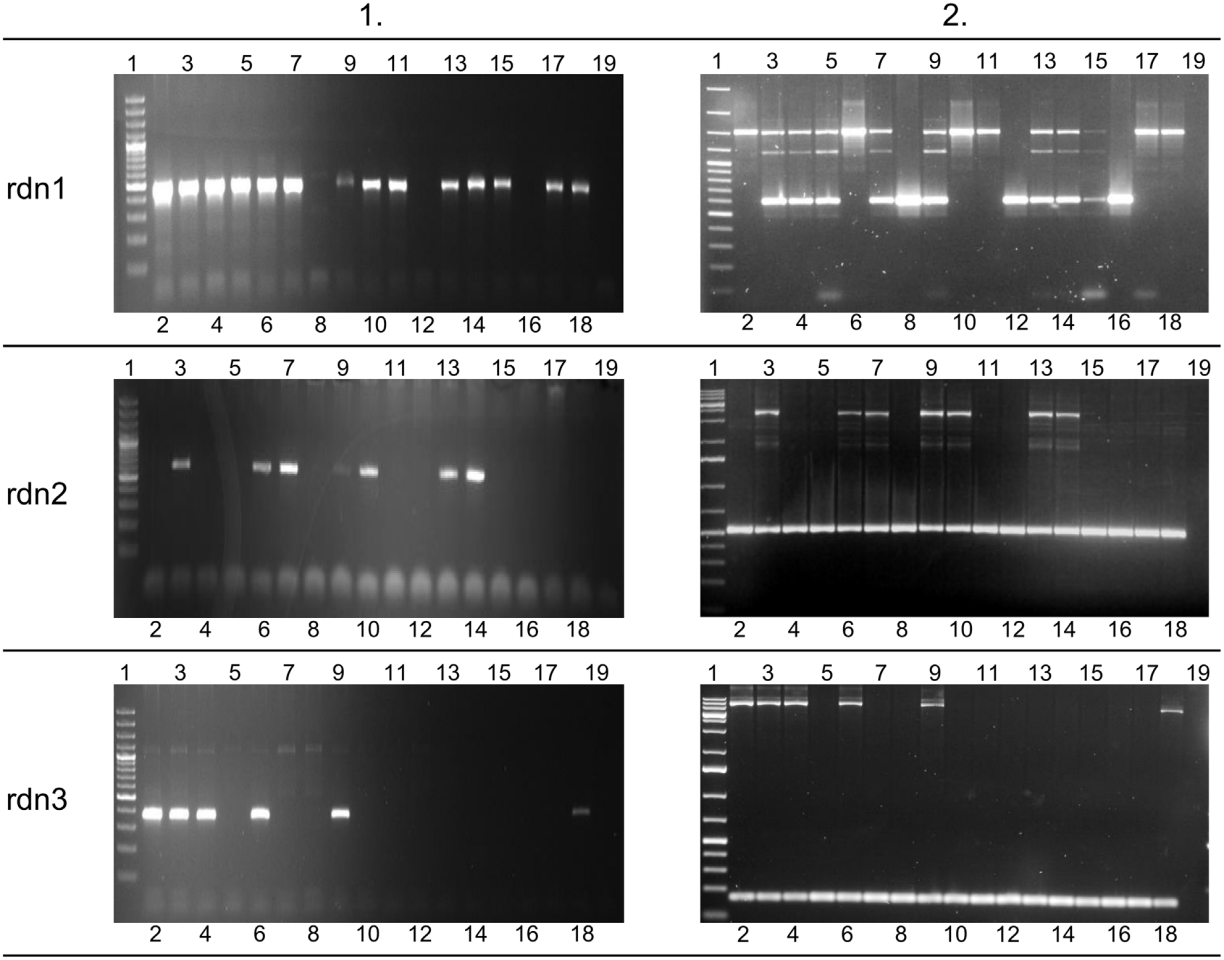
Agarose gel results for novel retrogenes. Left side of figure (column 1) represents presence or absence of novel retrogenes in 17 examined genomes. Agarose gels on the right side of figure (column 2) show PCR products corresponding to the region of deletion. This indicates homo- or heterozygotic character of 17 studied individuals. Lane 1 –GeneRuler 100 bp Plus DNA Ladder or GeneRuler 1 kb DNA Ladder (Thermo Scientific); lanes 2–18 –genomic DNA templates (see S1 Table for details), lane 19 –negative control (water instead of DNA).

To confirm the identified retrocopies and their deletions in some individuals, we performed PCR on 17 human genomes supplied by the 1000 Genome Project (Coriell Cell Repositories). Individuals were selected based on previous bioinformatic analyses, and each originated from a different population. Using primers from identified retrocopy sequences, we confirmed the existence of four retrocopies (rdn1–rdn4). Retrocopy rdn5 contains a large number of repetitive sequences. Therefore, we could not obtain a product specific enough to confirm the presence of this particular retrocopy: it was excluded from further studies. However, we were able to prove the deletions of retrocopies rdn1 to 3 in some individuals. Apparently, retrocopy rdn4 was present in all investigated genomes. In addition, utilizing primers designed from the flanking regions of the deletion, we confirmed the size of the deleted regions (Table 3), as well as the homo- and heterozygosity of the studied individuals (Fig 8).

None of discovered retrocopies has a conserved open reading frame, which may suggest that they are non-functional. Using standard PCR in a pooled cDNA library, we searched for the expression of these retrocopies. We obtained an amplification product in only one retrocopy, rdn2. Its expression is also confirmed by three EST sequences deposited in the dbEST (GenBank: DA381977, AV702346, AV629627).

## Discussion

Retroduplication variations (RDVs) are a class of genomic sequence polymorphisms associated with the presence or absence of retrocopies in individual genomes. Until recently, our knowledge of the prevalence and significance of this phenomenon was limited. The advent of large-scale sequencing projects, such as The 1000 Genomes Project, allowed researchers to detect such polymorphisms on a massive scale, including both known and novel (not present in the reference genome) retrocopies. Particularly the latter class has drawn much attention, as their discovery is much more challenging than determining the presence or absence of an annotated retrocopy. Previous studies [14–16] focused on relatively recent retroduplication events, which is hardly surprising given that they are very informative on the history of human populations. Two of these studies were indeed able to reconstruct population phylogenetic trees from detected retroduplication variations. Identifying these novel retrocopies required mapping short reads to the genomic sequences spanning splice sites of their parental genes. However, not every novel human retroduplication polymorphism can be discovered in that way. For example, retrocopies that originated very early in the human lineage might have diverged too much from their parental genes to be mapped to their sequences. Similarly, some ancestral retrocopies may have been lost from some of the ancient genomes and are thus, very infrequent in contemporary human populations. As a result, they were not included in the human reference genome. Finally, some retrocopies may not be included in the reference genome, simply due to genome misassembly.

Using our two approaches, we were able to detect 193 RDVs (190 RDVs affecting annotated retrogenes and 3 RDVs of retrocopies absent from the reference genome). This is slightly less than the sum of all previous studies, but significantly more than in any of these studies individually (176 [15], 58 [14], and 91 [16]). However, due to a different approach, our results complement rather than overlap previous studies. While previous studies mainly focused on RDVs from recent retroposition events, our dataset revealed RDVs resulting from retrocopy deletion. We found orthologs for only 56 retrocopies missing in some human genomes. Nevertheless, we are confident that indels resulting from a deletion dominate in this set, since frequency of retrocopy absence is low for the majority of them; absence in more than 50% of individuals was observed in only a few of the retrogenes. For new retropositions, we would expect absence to be close to 100% of alleles in at least some populations.

In this study we developed an approach to detect and characterize hitherto undiscovered retroduplication variations in the human genome. To accomplish this, we de novo assembled the unmapped reads from 500 individuals whose genomes were sequenced in the 1000 Genomes Project. Contigs from different individuals were subsequently combined and assembled into novel genomic regions. Finally, genomic regions were filtered to exclude cross-species contaminations and sequences from alternative human genome assemblies (HuRef) and reference genome patches (GRCh37.p10). These filtered sequences were searched for retrocopies using the RetrogeneDB pipeline [19]. Using this strategy, we were able to discover 5 novel retrocopies, none of which were previously detected [14–16]. We experimentally confirmed the existence of the detected retrocopies and checked for their presence and absence in the human genomes from 17 different populations. The profile of retrocopy presence varied greatly between retrocopies, with some of them being ubiquitous and some detected in only few populations. Detected retrocopies showed significant divergence between their sequences and sequences of their parental genes, indicating an old retroposition event. For all of these retrocopies, we were able to find the orthologs in the chimpanzee or gorilla genome, confirming that they are, in fact ancestral retroduplications that were subsequently lost during human evolution.

The number of discovered novel retrocopies (i.e. not annotated on the reference genome) is much lower than in previous studies [14–16]. This is mostly due to the fact that the current human reference genome is of very high quality. As a result, most polymorphic retroduplications with sequences clearly distinguishable from their parental genes are simply present in the reference genome. Our results suggest that we have reached the limit where the reference genome contains almost the entire human retrogene repertoire, with the exception of recent retroduplications, for which obtaining the exact sequence from the genome sequencing data is not trivial, as they are extremely similar to their parental genes. This is certainly not the case for most sequenced genomes, and our approach can be used to complement the retrogene set in many low-coverage, draft euakryotic genomes, for which resequencing data is available. Our pipeline can also be potentially applied to detect retrocopies specific to genomes of ancient, extinct organisms, such as neanderthal [24] or woolly mammoth [25].

The role of retrocopy polymorphisms as markers for human population history is clearly established, but our findings suggest that they can also provide great insight into ongoing evolutionary processes. The availability of population RNA-Seq data from the Geuvadis Consortium [18] allowed us not only to study changes in retrocopy sequences and repertoire, but also consider their potential functional implications. We particularly focused on expressed retrogenes that are absent in the genomes of some individuals. RDVs seem to affect both expressed and non-expressed retrocopies indiscriminately, but their allele frequency for most deletions of expressed retrocopies is relatively low (< 2%), suggesting that they are new or slightly deleterious.

We experimentally confirmed the expression of 11 ancestral transcriptionally active retrogenes undergoing deletions. The frequency of their deletion is very low with the exception of retrogene retro_hsap_4873, which is absent in over 10% of alleles in African populations. The expression, ancient origin and low rate of deletion of the remaining 10 retrocopies may suggest some functionality, but further studies would be required to establish their function and the possible consequences of their deletion.

Numerous studies revealed a tendency of retrogenes to be expressed in the testis [9, 26]. It has been hypothesized that this specific transcription may be the result of the hypertranscription state observed in meiotic and postmeiotic spermatogenic cells [27]. Alternatively, retrocopies could be preferentially inserted into actively transcribed, and therefore open chromatin [28]. Since the retroposition occurs in the germ line, retrocopies may be primarily inserted into, or near to genes expressed in the germ line, which may enable or enhance their expression in testis. Another hypothesis links this testis-specific expression with an escape from the male meiotic sex chromosome inactivation [29]. To verify the tendency of retrocopies to be expressed in testis, we performed expression analysis on 16 cDNA libraries from various human organs. Our findings confirmed testis-specific expression of only one retrogene. This supports results from our previous studies, showing that many retrogenes have broad expression patterns [8]. However, the set of analyzed retrogenes is relatively small and may not be fully representative.

Apart from the presence or absence of expressed retrocopies, we also searched for more subtle differences in retrocopy expression levels between populations. Overall, we detected 9 retrogenes that undergo statistically significant differential expression. Similar to inter-population differences in RDVs frequencies, retrogene expression differences tend to coincide with geographical locations, with most differences occurring between African and European populations. Observed differences most likely arise from different frequencies of short sequence variants (SNPs and indels) in the regulatory sequences of the retrogenes studied, but we cannot exclude more complicated scenarios, such as variation in DNA methylation. At this point, we still know very little concerning the functional implications of discovered expression differences, and this will require further study.

## Materials and Methods

### Detecting RDVs of known retrocopies

The set of human retrocopies generated by us from the RetrogeneDB database [19] was used for the identification of RDVs from known retrocopies. Sequence variants detected for 1,092 individuals during Phase 1 of the 1000 Genomes Project [13] were downloaded and searched for long deletions (structural variants) that overlapped retrocopy loci using BEDtools [30]. Only deletions that resulted in the loss of at least 100 bp of the retrocopy sequence were reserved for further analysis. For each deletion its frequency was calculated in each of the 14 analyzed populations (ASW, CEU, CHB, CHS, CLM, FIN, GBR, IBS, JPT, LWK, MXL, PUR, TSI and YRI).

### Retrocopy conservation analysis

Orthologs of human retrocopies in 13 other eutherian species were identified using Amniota vertebrates PECAN [31] whole genome alignment from Ensembl release 73 [32], requiring a reciprocal overlap of at least 50% between retrocopy sequences from different organisms in the alignment. To be more specific, we used bx-python package to extract genome alignment blocks corresponding to retrocopies annotated in RetrogeneDB. BEDtools [30] software was then used to find sets of overlapping blocks that constituted orthologous groups of retrocopies. We considered a human retrocopy to be ancestral if it had an ortholog in at least one other primate species (chimpanzee, gorilla, orangutan, macaque or marmoset).

### De novo assembly of novel retrocopy sequences and indel site identification

To search for novel retrocopies in the human genome, short reads that failed to align to the human reference genome for 500 individuals representing all populations included in Phase 1 of The 1000 Genomes Project were downloaded. Short reads from each individual were separately assembled using SOAPdenovo [33] and only contigs with a minimal length of 500 bp were retained. Then, clustering on all the assembled contigs using CD-Hit-EST [34] was performed, requiring at least 95% identity over at least 70% of the sequence. For each cluster, consensus sequence using CAP3 [35] was calculated, obtaining the set of novel genomic regions. Sequences that showed similarity to human genome patches and alternative assemblies (GRCh37.p10, HuRef), as well as genomic contaminations from viruses, bacteria and parasitic organisms were excluded from further analysis. Finally, we applied the retrocopy identification pipeline from RetrogeneDB [19] to detect and characterize retrocopies in assembled genomic regions. The pipeline takes a very stringent approach to identifying gene copies that originated via retroposition. The retrocopy identification process is based on the alignment of the whole proteome to the genome, using LAST software [36]. Observed alignments must meet numerous criteria to be considered retrocopies, including a length of at least 150 bp on the genomic sequence, alignment identity and protein coverage greater than 50%, and the loss of at least 2 introns inferred from the alignment.

To extend contig sequences, unassembled BAC clones and fosmids were searched using BLAST [37]. Identified human BACs and fosmids were then aligned to chimpanzee and/or gorilla genomes to identify homologous sequences. Deletion sites in the reference genome were detected based on comparison of sequence and gene locations in human, chimpanzee and gorilla genomes (Fig 7).

### Novel retrogenes and indels confirmation

Novel retrogenes and indels were confirmed experimentally by PCR in the genomes of 17 individuals from various origins (S3 Table) from Coriell Cell Repositories. PCR was carried out using primers designed from the retrocopy sequence and sequences flanking the RDV.

### Expression pattern analysis

Expression of retrogenes was analyzed in Multiple Tissue cDNA Panels from Clontech—Human MTC Panel I and II (catalog no. 636742 and 636743 respectively). This set of tissues contained the following: heart, brain, placenta, lung, liver, skeletal muscle, kidney, pancreas, spleen, thymus, prostate, testis, ovary, small intestine w/o mucosal lining, colon and peripheral leukocyte cDNA.

### Standard PCR

All PCR primers used in this study were designed or verified using Primer-BLAST with the following parameters: primer melting temperature (T_m_) 55–60°C and GC content between 40% and 60% (S2 Table).

Standard PCR amplification was done using EconoTaq PLUS 2X Master Mix (Lucigen). The reaction was carried out in a total volume of 10 μl, containing 1 μl of DNA template (genomic DNA or cDNA), 1X EconoTaq PLUS Master Mix, and 1 μM of each primer (S2A and S2B Table). Thermal cycling conditions were as follows: 2 min at 94°C, followed by 40 cycles of 94°C for 30 sec, 55–60°C for 15 sec, 72°C for 1 min, and a final 5 min at 72°C and 4°C hold. PlatinumTaq DNA Polymerase (Life Technologies) was used for long-size products amplification. PCR mixes in 25 μl volumes contained: 1 μl of DNA template, 1X High Fidelity PCR Buffer, 0.2 mM of each dNTP, 2 mM MgSO4, 1 μM of each primer (S2C Table), and 1 unit of Platinum Taq High Fidelity Polymerase. PCR reactions were performed as follows: 2 min at 94°C, followed by 30 cycles of 94°C for 30 sec, 55–57°C for 30 sec, 72°C for 12 min, and a final 12 min at 72°C and 4°C hold.

Electrophoreses were done in 1.5% agarose gels containing GelRed (Biotium) in 1x TAE buffer. Half of the PCR reaction volume was used for gel electrophoresis. When additional or nonspecific products were observed, the expected size product was excised from the gel and purified using Gel Extraction Kit (Bio Basic INC). Re-PCR reaction was performed using the extracted DNA as the template and EconoTaq PLUS 2X Master Mix mentioned before. When sufficient product was obtained, the remaining half of the total reaction volume was purified with 5 μl mix of Exonuclease I and FastAP (Thermo Scientific). The samples were incubated at 37°C for 30 minutes, followed by enzyme deactivation at 80°C for 15 minutes.

Following enzymatic purification, the PCR products expected to confirm retrogene expression or presence of novel retrogenes in 17 human genomes were sequenced. Sequencing was performed with the Big Dye V3.1 Terminator Kit (Applied Biosystems) using forward and reverse primers designed for each retrogene (S2A and S2B Table) on an ABI Prism 3130xl machine (Applied Biosystems). Additionally, short PCR products corresponding to genomic loci with deletion (S2C Table) were purified and sequenced.

The BioEdit Sequence Alignment Editor [38] was used for parental gene, retrogene and sequencing result global alignment. Sequencing result specificity was verified by megaBLAST analysis.

### Real-time PCR

Real-time polymerase chain reaction was performed in Applied Biosystems QuantStudio 6 & 7 Flex Real-Time PCR System using Power SYBR Green PCR Master Mix (Applied Biosystems) in 40 cycles and with Tm = 60°C. GAPDH gene was used as an endogenous control. Results were analyzed using QuantStudio Real-Time PCR Software v1.0 (Applied Biosystems). The cut-off value for Ct (cycle threshold) was established as 32, based on previously published studies [8]. Additionally, results were exported into a RDML data format [39] and used for PCR efficiency estimation using LinRegPCR software [40]. An efficiency value over 1.8 was obtained for all retrogenes and considered as acceptable. The relative amount of each retrogenes against GAPDH was calculated using the equation 2^−ΔCt^×10^6^, where ΔCt = (Ct_retrogene_-Ct_GAPDH_).

### Differential expression analysis

In order to analyze retrocopy expression, RNA-Seq samples from 10 individuals for each of 5 populations (CEU, GBR, FIN, TSI, YRI) from the Geuvadis RNA sequencing project [18] were used, as well as data for 16 human tissues from the Illumina Bodymap 2.0 project. Tophat [41], provided by Ensembl release 73 annotations, was used to align short reads from each sample to the reference sequence consisting of GRCh37.p10 genome assembly and de novo assembled novel retrocopy sequences. Retrocopy expression estimation was based on the number of read pairs that mapped concordantly and uniquely (mapping quality equal or greater to 50) to each locus. Only retrocopies with a normalized expression value of at least 1 RPM (reads per million mapped reads), were considered to be expressed. Furthermore, the DESeq2 package [23] was used to search for significant differences in retrocopy expression between analyzed populations, requiring at least a 2 fold expression change and adjusted p-value less than 0.05. Loci with low coverage (mean number of reads per biological replicate less than 50) were excluded from the analysis.

## Supporting Information

S1 Fig Agarose gel results showing PCR products obtained from 16 cDNA templates. Black arrows indicate PCR products of retrogenes undergoing deletions in various human populations (A-J). Lane 1 –GeneRuler Low Range DNA Ladder (Thermo Scientific); 2 –Heart; 3 –Brain; 4 –Placenta; 5 –Lung; 6 –Liver; 7 –Skeletal Muscle; 8 –Kidney; 9 –Pancreas; 10 –Spleen; 11 –Thymus; 12 –Prostate; 13 –Testis; 14 –Ovary, 15 –Small intestine 16 –Colon; 17 –Leukocyte; NTC—No template control (water instead of cDNA).
(TIF)

S1 Table Frequencies of retrogenes loss in human populations. (XLSX)

S2 Table PCR primers used for experimental validation. PCR primers used in: A. retrogenes expression analysis, B. detection of novel retrogenes in 17 human genomes and expression analysis, C. detection of novel retrogene insertion sites in the reference genome. * Set of primers used also in real-time PCR. **Second pair of primers for retrogene retro_hsap_2011 was used to confirm a longer splice variant (TPM3P9-002, ENST00000424846). ***Product size with/without deletion.
(XLSX)

S3 Table Human genomes from Coriell Cell Repositories examined in order to confirm novel retrogenes and identify indels. (XLSX)
