# Supplementary figures and images for: Inter-population Differences in Retrogene Loss and Expression in Humans
Source: PLoS Genet. 2015 Oct 16;11(10):e1005579. doi: 10.1371/journal.pgen.1005579 (PMC4608704; doi:10.1371/journal.pgen.1005579)

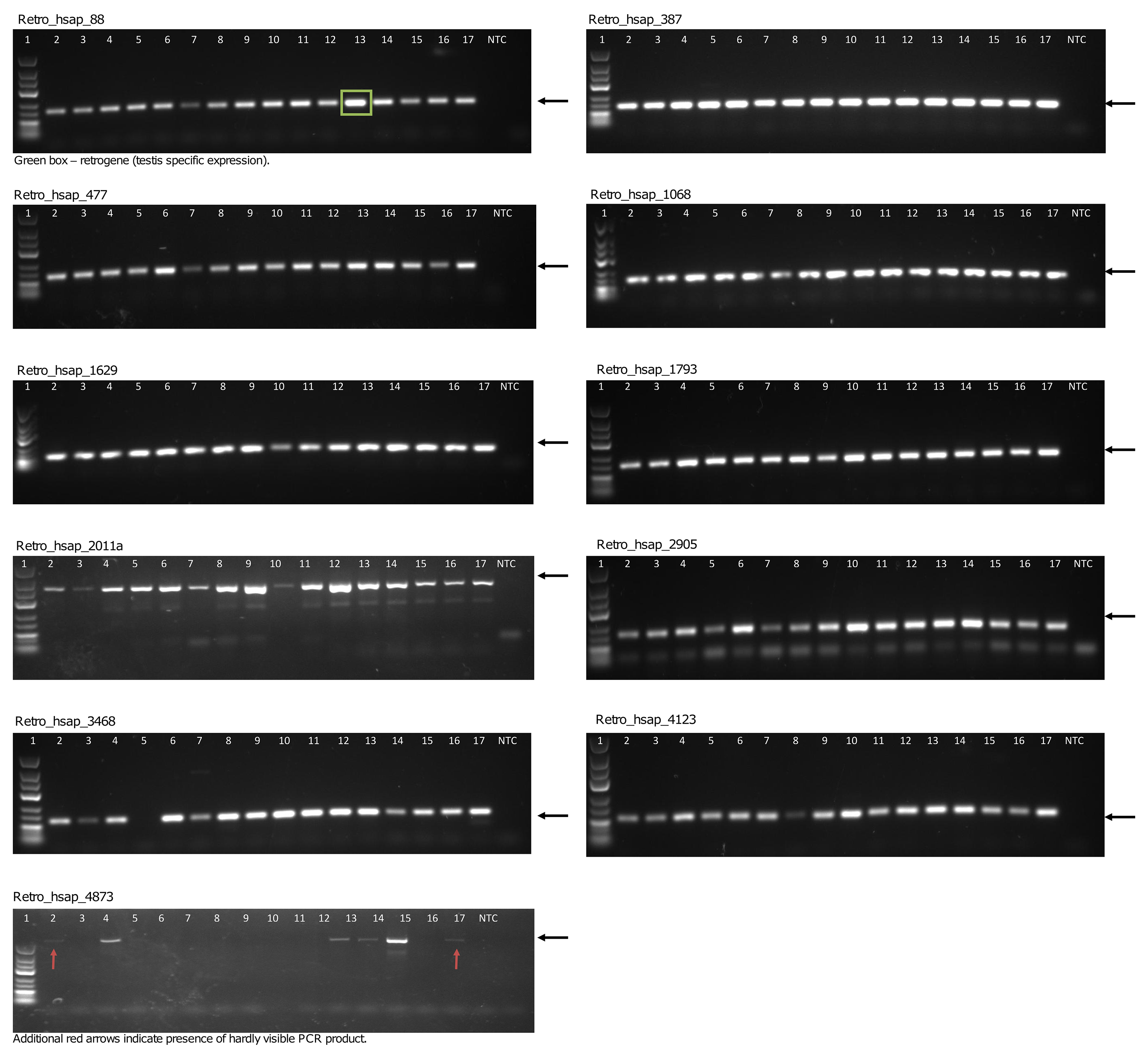

Supplement: S1 Fig — Black arrows indicate PCR products of retrogenes undergoing deletions in various human populations (A-J). Lane 1 –GeneRuler Low Range DNA Ladder (Thermo Scientific); 2 –Heart; 3 –Brain; 4 –Placenta; 5 –Lung; 6 –Liver; 7 –Skeletal Muscle; 8 –Kidney; 9 –Pancreas; 10 –Spleen; 11 –Thymus; 12 –Prostate; 13 –Testis; 14 –Ovary, 15 –Small intestine 16 –Colon; 17 –Leukocyte; NTC—No template control (water instead of cDNA). (TIF) [file pgen.1005579.s001.tif]
